# Perceptions and Attitudes Toward the Use of a Mobile Health App for Remote Monitoring of Gingivitis and Willingness to Pay for Mobile Health Apps (Part 3): Mixed Methods Study

**DOI:** 10.2196/26125

**Published:** 2021-10-05

**Authors:** Guy Tobias, Harold Sgan-Cohen, Assaf B Spanier, Jonathan Mann

**Affiliations:** 1 Department of Community Dentistry Faculty of Dental Medicine The Hebrew University-Hadassah School of Dental Medicine Jerusalem Israel; 2 Department of Software Engineering Azrieli College of Engineering Jerusalem Israel

**Keywords:** mHealth, public health, oral health promotion, gum health, willingness to pay, willingness to use, willingness, perception, attitude, mouth, oral health, dentist, app, monitoring, mixed method

## Abstract

**Background:**

Gum infection, known as gingivitis, is a global issue. Gingivitis does not cause pain; however, if left untreated, it can worsen, leading to bad breath, bleeding gums, and even tooth loss, as the problem spreads to the underlying structures anchoring the teeth in the jaws. The asymptomatic nature of gingivitis leads people to postpone dental appointments until clinical signs are obvious or pain is evident. The COVID-19 pandemic has necessitated social distancing, which has caused many people to postpone dental visits and neglect gingival health. iGAM is a dental mobile health (mHealth) app that remotely monitors gum health, and an observational study demonstrated the ability of iGAM to reduce gingivitis. We found that a weekly dental selfie using the iGAM app reduced the signs of gingivitis and promoted oral health in a home-based setting.

**Objective:**

The aim of this mixed methods study is to assess perceptions, attitudes, willingness to pay, and willingness to use an mHealth app.

**Methods:**

The first qualitative phase of the study included eight semistructured interviews, and the second quantitative phase included data collected from responses to 121 questionnaires.

**Results:**

There was a consensus among all interviewees that apps dealing with health-related issues (mHealth apps) can improve health. Three themes emerged from the interviews: the iGAM app is capable of improving health, the lack of use of medical apps, and a contradiction between the objective state of health and the self-definition of being healthy. Participants were grouped according to how they responded to the question about whether they believed that mHealth apps could improve their health. Participants who believed that mHealth apps can enhance health (mean 1.96, SD 1.01) had a higher willingness to pay for the service (depending on price) than those who did not believe in app efficacy (mean 1.31, SD 0.87; *t*_119_=−2417; *P*=.02). A significant positive correlation was found between the amount a participant was willing to pay and the benefits offered by the app (rs=0.185; *P*=.04).

**Conclusions:**

Potential mHealth users will be willing to pay for app use depending on their perception of the app’s ability to help them personally, provided they define themselves as currently unhealthy.

## Introduction

### COVID-19 and Gingivitis

Gingivitis [1] is a reversible gum inflammation, characterized by red, swollen, and bleeding gums. More than 80% of the global adult population is affected by gingivitis periodically [2]. Usually, it heals within 10 days of at-home oral hygiene practices that include twice-daily brushing and interproximal cleaning using dental floss, toothpicks, and mouthwash [3]. In dental clinics, gingivitis is usually treated in a single cleaning session where plaque and calculus are removed. Untreated gingivitis develops into periodontitis [4,5], the irreversible stage of periodontal disease, in which bacterial toxins and the immune response to them destroy the tissues that support the teeth. Treatment for periodontitis is complex and requires multiple appointments and sometimes surgical intervention. As the disease progresses, teeth may become mobile or lost [6].

Most people recognize the early signs of gingival inflammation, namely bleeding while brushing or eating something hard, an unpleasant odor, or swollen gums [1]. However, because gingivitis is not painful, appointments tend to be postponed until the disease is more advanced [3]. Currently, we are in the midst of a major public health crisis. The COVID-19 pandemic [7] has necessitated new behaviors and rules to limit the spread of the infection, such as wearing masks, hand hygiene, and maintaining 2 m of space between people (social distancing).

Dentistry is a field with close contact between patients and the clinical team, with a high risk of infection transmission [8]. Keeping in mind that most people only seek dental treatment when experiencing pain, we assume that because of the current restrictions, people will come even less, resulting in oral health deterioration, especially in those with gingivitis.

### Remote Patient Monitoring

Remote patient monitoring (RPM) [9] is a term describing technologies that enable patient-therapist interaction without a physical meeting, for example, when the patient is at home and the care provider is elsewhere. RPM eliminates problems of geographical distance, improves access to treatment, and reduces the indirect costs of traditional clinical treatment, such as travel time, fuel, and parking. RPM improves treatment access to individuals living far from medical centers and to populations with limited mobility [10]. Furthermore, RPM encourages people to seek treatment for nonurgent issues that tend to be postponed because of travel and time issues [11].

Incorporating RPM in the management of chronic diseases significantly improves patient quality of life, as less time is spent in the doctor’s office while maintaining contact and follow-up [12]. RPM is delivered using advanced technologies that quickly and effectively detect deterioration of the patient’s condition and update the treating staff and the patient. Early warning reduces the number of hospitalizations and hospitalization days and improves quality of life [13]. The components [9] of RPM are (1) disease-specific sensors that monitor physiological and pathological changes; (2) storage of relevant information accessible to attending physicians; (3) an encrypted server where personal information is stored securely; and (4) diagnostic tools, software that facilitates information retrieval and helps develop therapeutic recommendations based on the patient’s data.

In dentistry, as far as we know, no work has been done regarding RPM, and there are currently no technologies for remote oral health monitoring.

### iGAM App

iGAM [14] is a dental mobile health (mHealth) app that remotely monitors gingival health, and our previous article described the development process followed by a pilot study to evaluate the acceptance of remotely monitoring gum health.

The iGAM mHealth app has three main features:

- A self-completion questionnaire that deals with knowledge and attitudes toward oral hygiene habits.
- Text accompanied by illustrations describing brushing techniques as well as short articles about the importance of maintaining oral health in general and during pregnancy, the implications of smoking on gum health, and the connection between gum diseases and general health.
- Feature for self-photography of the gums using the rear camera of the smartphone, that is, the *dental selfie*.

We performed an 8-week observational study, with 126 participants divided into 2 groups. The first group photographed their gums weekly, and the second group took only 2 pictures, one at the start of the study and the second 8 weeks later. We found that a weekly dental selfie using the iGAM app reduced the signs of gingivitis and promoted oral health. These findings were published in part 2 of this series of articles [15].

### Health Belief Model and Willingness to Pay for mHealth Apps

The fact that an mHealth app is capable of improving health does not guarantee that the public will use it. Some degree of health literacy and a mobile device with advanced technological capabilities, availability of storage space, and money are needed. The Health Belief Model (HBM) [16] is a psychological model developed in 1950 by social psychologists Hochbaum, Rosenstock, and Kegels, which tries to explain and predict the adoption of health behaviors by focusing on the attitudes and beliefs of the individual.

The HBM has five content categories [17]:

- Perceived susceptibility—understanding the possibility of developing a specific disease. Those who estimate that the likelihood of getting sick is low deny the possibility that they will become unwell, whereas those with a high degree of sensitivity estimate that they are in real danger of getting sick.
- Perceived severity—understanding the severity and seriousness of a specific disease and its consequences. This category reflects an individual’s beliefs regarding the difficulties that illness may cause, such as pain, discomfort, and financial burden.
- Perceived benefits—believing that recommended health behavior will be beneficial by preventing the disease or reducing its effects.
- Perceived barriers—the costs of or obstacles to performing the recommended behavior, including tangible costs (eg, time, money, availability, and skill acquisition) and the psychological costs associated with performing the behavior (eg, pain, feeling anxious, pessimistic, and embarrassed). A low perception of barriers increases the likelihood of adopting such behavior.
- Cues to action—the circumstances that inspire the readiness to act.

The model assumes that people take action when they perceive a threat to their health and consider whether the benefits of a new behavior are greater than the obstacles to performing it. The more benefits the individual believes there are from the behavior, the greater the chance of adopting it [18].

The principles of the HBM have been used as a conceptual basis for many studies. It should be noted that many studies address only some of their components.

Willingness to pay (WTP) [19] describes the maximum amount a consumer is prepared to pay for a particular service or product. It was found that the more the consumer perceives that the technology promotes health, the more they will be inclined to pay for it. A study [20] examining the willingness of young Iranians to pay higher prices for organic food products found that perceived benefits and barriers were significant predictors of the WTP. Those believing that consumption of organic food was beneficial were more willing to purchase organic food at higher prices. WTP research has two main branches [21]: (1) RP-revealed preference, where the WTP is estimated by real market analysis, and (2) SP-stated preference, where the WTP is estimated by consumer responses to hypothetical scenarios in which consumers are asked to indicate their preferences. The second method is used when it is impossible to obtain real data about consumer preferences, for example, when determining the demand for a new product. Wong et al [22] used a cross-sectional study with 1159 participants and asked about their WTP based on the HBM model. They found that most participants described the observed benefit from a corona vaccine as the main reason they would be willing to pay more for it.

The aim of this mixed methods study is to use qualitative and quantitative methods to examine attitudes and acceptance of mHealth apps in general and an app for remotely monitoring gingivitis (iGAM) in particular. Another goal is to examine the WTP for mHealth apps as a way to characterize responsiveness to mHealth app use.

## Methods

### Overview

This mixed methods study was conducted between March 2020 and July 2020 at the Department of Community Dentistry Faculty of Dental Medicine, the Hebrew University, Hadassah School of Dental Medicine. The protocol was approved by the Hadassah Research Ethics Committee (institutional review board, 0212-18-HMO), and written informed consent was obtained from all participants. There was no payment for participation. The study was conducted in two phases: a qualitative study based on semistructured interviews that reached saturation and highlighted the need for further data that was gathered in a quantitative study based on answers to questionnaires.

### Qualitative Study Design and Data Collection

A total of 10 individuals from 126 participants in the 8-week observational study were randomly selected. They were asked via an SMS text message sent through the app whether they would participate in a 50- to 60-minute phone interview (the interview could not be done in person because of COVID-19 social distancing restrictions) to discuss their experience with the iGAM app and their thoughts regarding mHealth apps. Approximately 3 days later, a follow-up SMS text message was sent through the app. Of the 10 people, 8 agreed to the interview (one did not reply and one had schedule limitations). The semistructured interview was developed with guidance from two qualitative research methodology experts (Multimedia Appendix 1). The questions were divided into four sets: (1) opening questions—Tell me about yourself, What do you expect from your cellular phone? Describe your cellular phone use, etc; (2) questions regarding cellular apps in general and medical apps in particular: Describe the apps currently on your phone, How often do you use cellular apps? What sort of mHealth apps are you familiar with? In your opinion, what is the purpose of using mHealth apps? (3) Questions related to iGAM mHealth: describe your expectations of the app before the study, describe your experience using the app, do you think that using the app improved your oral health? In your opinion, who would this app help most? (4) Personal information, age, address, and occupation.

The interviews were audio recorded and transcribed using Microsoft Word 2016 (Microsoft Corporation). The primary author performed a qualitative data analysis. Each interview was read several times to identify the codes. The list of codes was grouped into categories based on content similarity, determined by counting the frequency that the interviewees talked about them. Examination of the categories enabled the identification of themes. At the end of the process, all authors reviewed the themes that emerged from the interviews.

### Quantitative Study Design and Data Collection

On completion of the observational study, questionnaires were sent to all 126 participants via the iGAM app, and an SMS text message was sent. The participants were told that they were not obligated to answer the questions and that their answers needed to be submitted within 3 weeks. A second SMS text message reminder was sent a week before the deadline. The questionnaire included 17 statements (Multimedia Appendix 2) on mHealth apps in general. The participant had to circle the response by matching their points of view. The statements were divided into two sets. The first set dealt with (1) beliefs about the ability of mHealth to improve health; (2) perceptions regarding the ability of mHealth to effectively monitor blood pressure, obesity, physical fitness, and oral health; (3) attitudes regarding searching for medical information on the internet; (4) personal use of medical apps; (5) statements about personal perceptions of health and state of health; and (6) reasons for not using mHealth apps. The second set dealt with a WTP for medical apps with costs between US $15 and US $150.

### Statistical Methods

Data were analyzed using SPSS Statistics software version 27.0. The significance level was set at a *P* value of .05. *t* tests for independent samples were used to examine WTP. A Pearson correlation was conducted to examine the relationship between the tendency to use apps and the WTP depending on price. Spearman correlations were conducted to examine the relationship between the amount the participant is willing to pay for an app with optimal functions and the tendency to use the apps and WTP according to the app features. All independent variables were included in the enter regression test.

## Results

### Qualitative Results

Eight interviews were conducted after 10 participants in an 8-week study were asked if they were willing to be interviewed. One interviewee did not respond to the two requests, and the second interviewee canceled at the last minute and was not interested in rescheduling. Interviews ranged from 32.25 to 55.75 minutes, with an average of 46.25 (SD 2.5) minutes. Analysis of the interviewees revealed that one is a physician aged 33 years with 3 years of experience. Two work as nurses, one aged 41 years with 10 years of experience and the other aged 30 years with 4 years of experience. Three were students, one was a fourth-year dental student aged 26 years and two were first-year biology students aged 25 and 23 years. One interviewee aged 32 years worked as a computer engineer with 5 years of experience, and one interviewee was an electronic technician aged 36 years with 9 years of experience.

A total of three main themes emerged from the interviews: (1) the iGAM app is capable of improving health, (2) lack of use of medical apps, and (3) a contradiction between the objective state of health and self-definition of being healthy.

#### The iGAM App Is Capable of Improving Health

There was a consensus among all interviewees that apps dealing with health-related issues (mHealth apps) can improve health; 2 participants added that mHealth apps may be able to improve access for those in remote areas and be beneficial. An analysis of interview content revealed that most interviewees considered health apps to be desirable and be able to improve the provision of health care by better allocation of resources:

In my opinion, this device can alert and improve, this device can say here it’s time to go to the dentist. I think it’s something that is very very good, it’s first of all will improve the patient’s health.

I would like to say that this app is good, it will serve both doctors and patients, both in Israel and in third world countries, as I told you, with low medicine, even in countries with advanced but expensive medicine, in the end it is in everyone’s interest.

I believe this app will have an impact on better health products.

#### Lack of mHealth App Use

When questioned about apps installed in addition to those that came with the device, apps from four content worlds arose: (1) social apps (WhatsApp, Facebook, and Instagram); (2) apps related to household management, bank account management, payments, deliveries, and orders from supermarkets; (3) apps related to leisure: Netflix, books, holiday booking, etc; (4) and apps for editing videos and photos. None of the participants installed additional medical apps on their device, and they did not use the apps that were already on the phone (eg, the pedometer).

When asked why there were no medical apps for them, 2 participants answered that they had technical limitations of phone memory space, and the remaining 6 explained that they had no need for a medical app as they were healthy:

I have WhatsApp, I have a camera, Facebook, a bank account, Maccabi, Wizz, booking, cheap vacations, photos.

I have no medical apps, other than what I got with the phone, pedometer because I do not need and I have no place on the phone.

No I do not need; I am healthy.

I do not use for example, speaking of blood pressure, once I measure myself sporadically and not consistently and once I understand that I do not need it because I am a healthy person, it is quite difficult to say that using the phone would improve my blood pressure, because I am healthy And feel fine.

#### The Contradiction Between the Objective State of Health and the Self-definition of Being Healthy

When the interviewees were asked about their state of health, they all said that they were healthy. When asked to expand on the subject, their perception of being healthy may not be accurate, 3 reported uncontrolled hypertension, 2 reported episodes of high blood glucose, and 1 reported frequent headache:

I’m healthy. I’m healthy, uh, my blood pressure occasionally jumps but it’s normal.

I sometimes feel headaches, occasionally I also get sugar, it does not say that I am sick.

No, I would not say I have a medical problem, I measure my blood pressure, and sometimes there are jumps, it does not mean it is unbalanced.

### Quantitative Results

#### Descriptive Statistics

Of the 126 participants asked to fill in the questionnaire, 121 responded positively. The 5 remaining individuals were contacted again (through the app) and did not reply. Of the 121 respondents, 91 (75.2%) were male; the age range was 19-66 years, with an average of 27.65 (SD 8.93) years; and the most common occupation was *student* (53/121, 44.2%). The results were analyzed by dividing the respondents into 2 groups based on their response to the yes/no question about the belief that mobile apps can improve health. A total of 13.2% (16/121) individuals were in group 1 and “lacked faith that apps can improve health,” and the remaining 86.8% (105/121) were in group 2 and “believed that mobile apps can improve health.” Most of the members of both groups responded that they first seek medical information on the web (12/16, 75% vs 90/105, 85.7%) and then go to a physician (11/16, 69% vs 63/105, 60%; group 1 versus 2, respectively). In addition, most members of both groups replied that they do not use mHealth apps to improve their health (14/16, 87% vs 70/105, 66%); however, if they had a long-term illness and the app associated with their illness was free, they would download it to their mobile device (12/16, 75% vs 97/105, 92.4%, group 1 versus 2, respectively). If they had to pay 50 NIS (currency of Israel–the New Israeli Shekel [NIS] $1=approximately 3.5 NIS) on a one-time basis, most of the group that did not believe that apps can improve their health would not buy it (9/16, 56%) and if it cost 500 NIS (US $150), none of this group would purchase it. Overall, 55.2% (58/105) of the group that believed that mobile apps can improve health stated that they would pay 50 NIS (US $15) for an app but not 500 NIS (89/105, 84.8%). When given the option to purchase an app that is able to (1) detect a health condition, (2) offer a personalized treatment plan, and (3) inform your personal physician when problems occur, 44% (7/16) versus 46% (48/105) of participants from group 1 and 2, respectively, stated that they would be willing to pay the maximum amount asked: *100 NIS*. Most participants believed that apps can be effective in obesity monitoring (13/16, 81% vs 100/105, 95%) and fitness monitoring (11/16, 69% vs 96/105, 91%) but not for monitoring hypertension (10/16, 62% vs 85/105, 80.9%) in group 1 versus 2, respectively. In group 2, 88.6% (93/105; those who believed that mobile apps can improve health) believed that apps can effectively monitor oral health versus 44% (7/16) of those in group 1 (those who lacked faith that apps can improve health; Multimedia Appendix 2).

To understand the significance of our findings and based on consultations with two experts in qualitative research methods, we used the responses (on a 4-point scale ranging from do not agree to completely agree) to sets of statements to determine the variables “Willingness to pay depending on price” and responses of agree or disagree to “Tendency to use applications” (on an 8-point scale). (1) “Willingness to pay depending on price,” based on responses to statements: If I were sick with a chronic illness, I would download a free medical app related to the disease; If I were sick with a chronic illness, I would download a 50 NIS (one-time fee) medical app related to the disease; If I were sick with a chronic illness, I would download a 500 NIS (one-time fee) medical app related to the disease. (2) “Tendency to use applications,” based on responses to statements: I believe that cell phone apps can improve health; When I need medical information about myself, I search the internet first; I use mobile applications to improve my health; If I were sick with a chronic illness, I would download a free medical app related to the disease; I have an interest in medical apps: Hypertension, Obesity, Fitness, Oral health.

The responses to the research variable ”Willingness to pay depending on price“ were given a score from 0 (do not agree) to 4 (completely agree), and the average score was 1.87 (SD 1.02). One point was given for each response of *agree* to the statements relating to the research variable ”Tendency to use applications,“ creating a score between 0 and 9. The average score was 6.02 (SD 1.44). The answers to the question, “If I were sick with a chronic illness I would pay a maximum amount of NIS (one-time fee) for an optimal medical app,” describing an ideal medical app was worded as follows: if you were diagnosed with a specific chronic disease and there was an app could monitor your illness and provide information about the stage of the disease you were in, recommend a reliable treatment plan, and contact your treating physician when changes in your health occur, ie, an optimal app—the maximum I would pay (one-time fee) is (a)10 NIS; (b)50 NIS; (c)100 NIS; and (d) I have no interest in this type of app—ranged from 0 (no interest in this type of app) to 4 (willing to pay 100 NIS), with an average of 2.82 (SD 1.30; Table 1).

**Table 1:** Means and standard deviations of the study variables.

Research variable	Value, mean (SD)
Willingness to pay depending on price	1.02 (1.87)
Willingness to pay for an optimal app	2.82 (1.30)
Tendency to use app	6.02 (1.44)

#### Analytical Statistics

**Figure 1:**
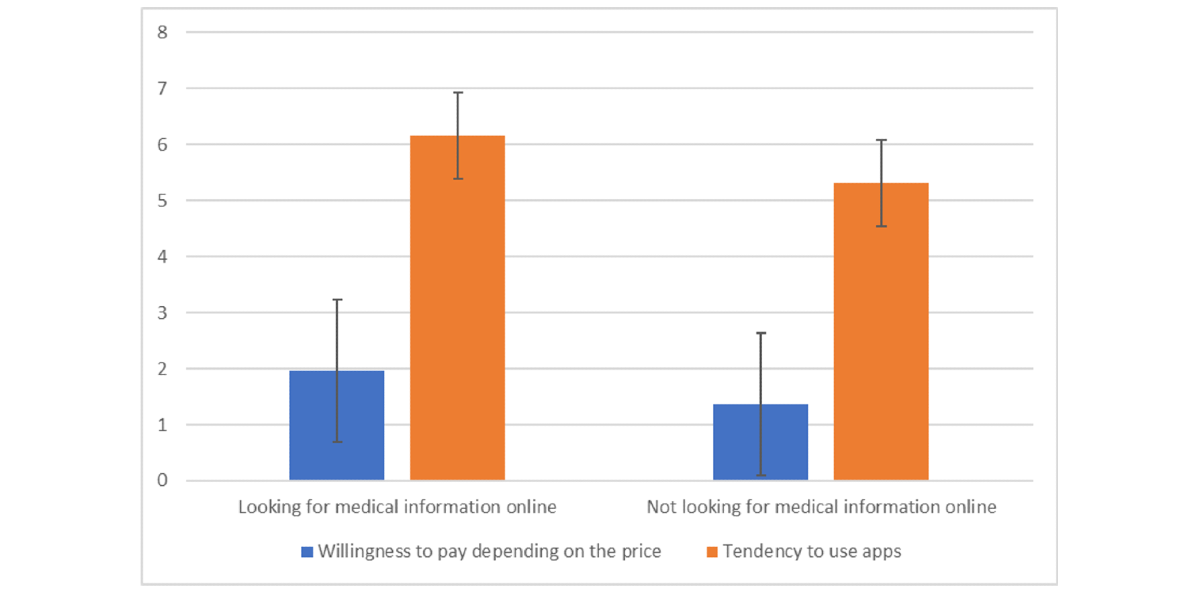
Average positive responses based on seeking or not seeking medical information on the internet.

Participants responding positively to the question, “when I need medical information about myself, I use the internet first” (mean 1.97, SD 1.06) showed a significantly greater tendency to use mHealth apps than those responding differently (mean 1.37, SD 0.60; *t*_119_=−2.407; *P*=.02; Figure 1). Furthermore, participants who first searched for medical information on the internet (mean 6.16, SD 1.48) showed a significantly higher WTP for an app than those who did not search for information on the internet (mean 5.31, SD 0.95; *t*_119_=−2.372; *P*=.02; Figure 1).

**Figure 2:**
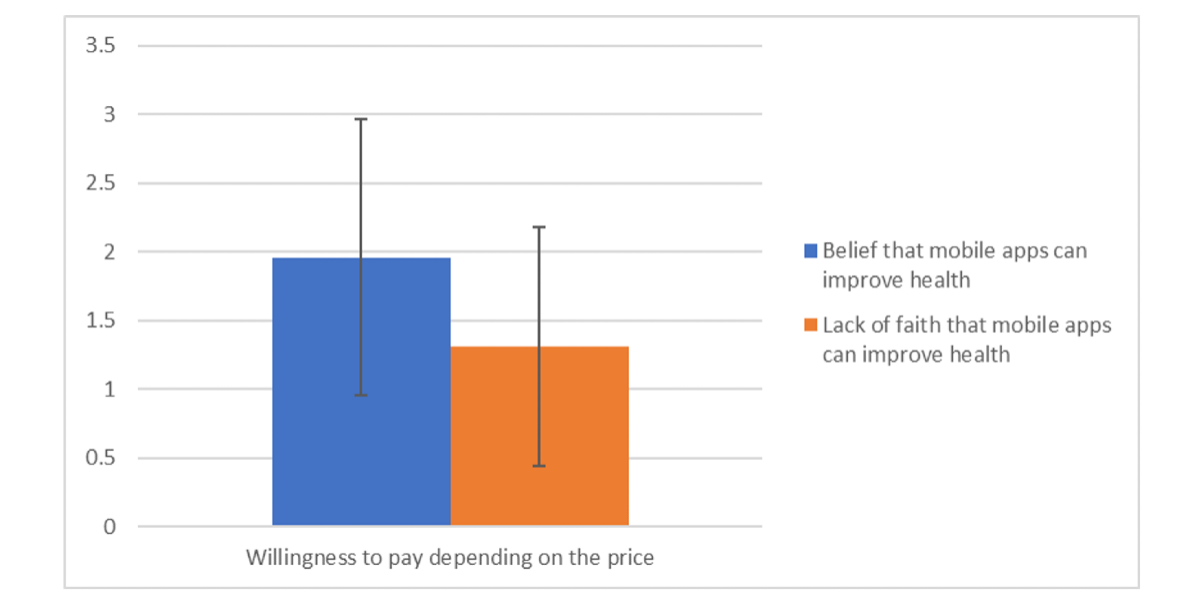
Willingness to pay depending on price according to the belief or lack of belief that mobile apps can improve health based on average positive responses.

Participants who believed that mHealth apps can improve health (mean 1.96, SD 1.01) were significantly more willing to pay for an app (depending on price) than those who did not believe in the efficacy of apps (mean 1.31, SD 0.87; *t*_119_=−2.417; *P*=.017; Figure 2).

**Figure 3:**
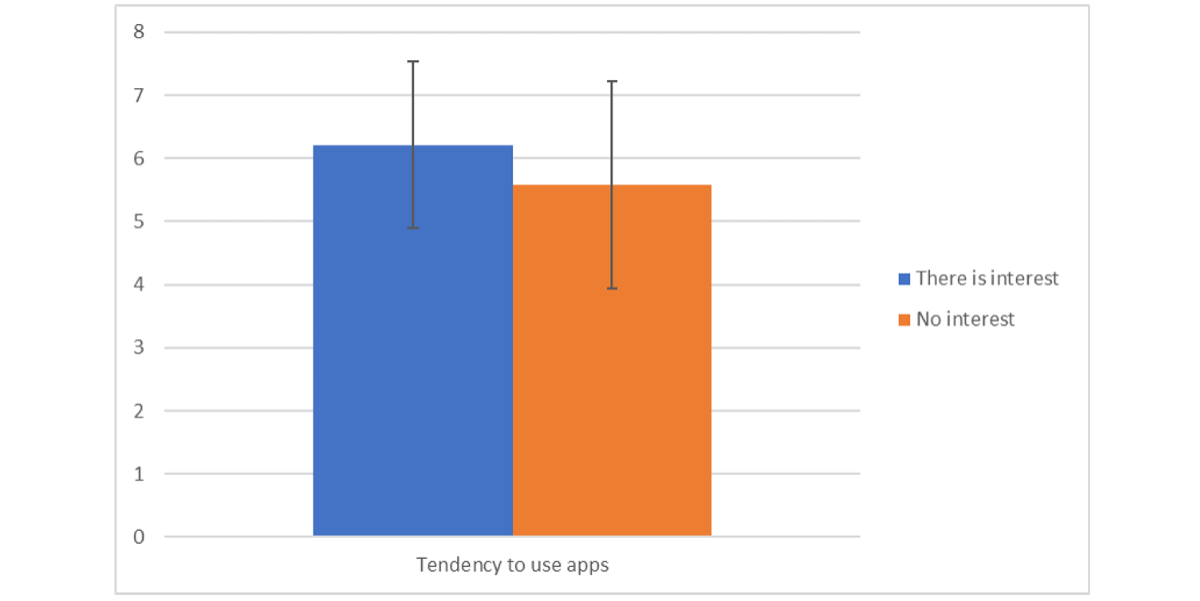
Tendency to use apps according to interest or no interest in paying based on average positive responses.

A significant positive relationship was found between the tendency to use apps and WTP depending on price (r=0.442; *P*<.001). In addition, individuals interested in paying for apps (mean 6.21, SD 1.32) had a significantly greater tendency to use apps than those who have no interest in paying (mean 5.58, SD 1.64; *t*_55,416_=−2.035; *P*=.047; Figure 3).

A significant positive relationship between the amount the participant was willing to pay for an optimal app and (1) the propensity to use apps (rs=0.185; *P*=.04) and (2) the WTP depending on price (rs=0.478; *P*<.001) was found. The regression test for predicting the WTP for an optimal app showed statistical significance (*F*_1,119_=207.272; *P*<.001) with an explained variance of 63.5%. Predicting a tendency to use apps was significant (*P*<.001); that is, the higher the tendency to use apps and the WTP depending on price, the higher the amount the participant is willing to pay for an optimal app.

**Figure 4:**
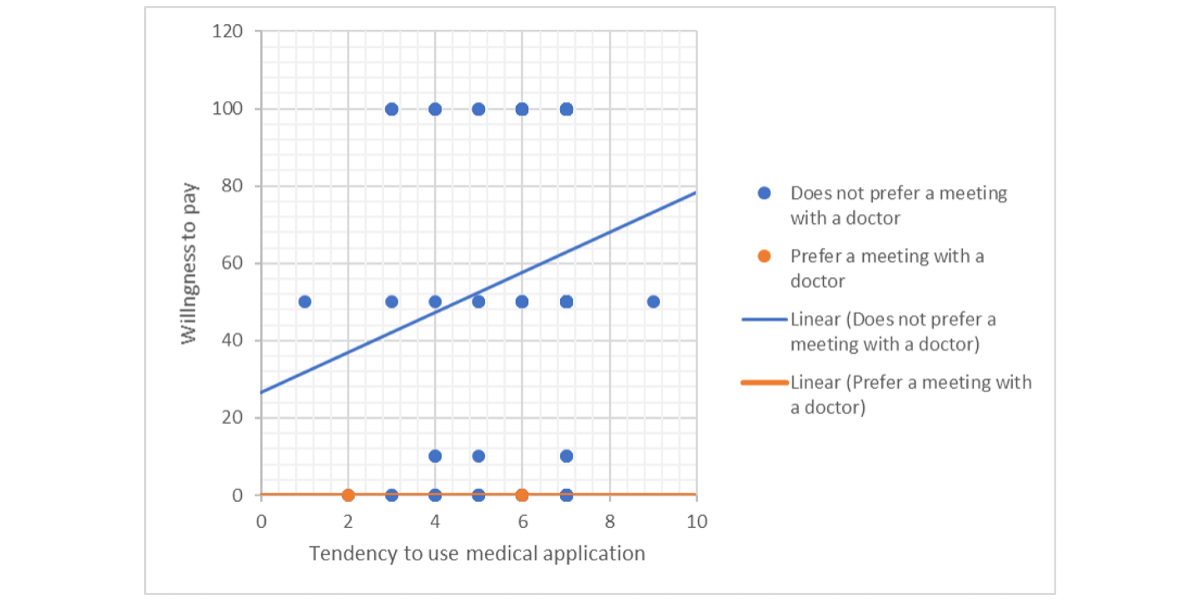
Tendency to use a medical app depending on willingness to pay. X axis: average positive responses to calculating the variable, “Tendency to use medical application.” Y axis: price (in New Israeli Shekel).

The question, “If you answered that you have no interest in medical applications, what is the reason for this?” examined the reasons for the lack of interest in using mHealth apps. The regression test showed statistical significance in predicting the use of available apps. The predictive variable with significance was “preference for an appointment with a doctor versus a lack of preference for an appointment with a doctor,” and participants who preferred to see a physician were less likely to use medical apps compared with those with the opposite preference (Figure 4).

The predictive variable, “I’m healthy so I do not need medical apps / other, and I have / I have no interest in paying,” was found to be statistically significant (*P*<.001). Participants who stated that they were healthy and did not need medical apps had no interest in payment.

**Figure 5:**
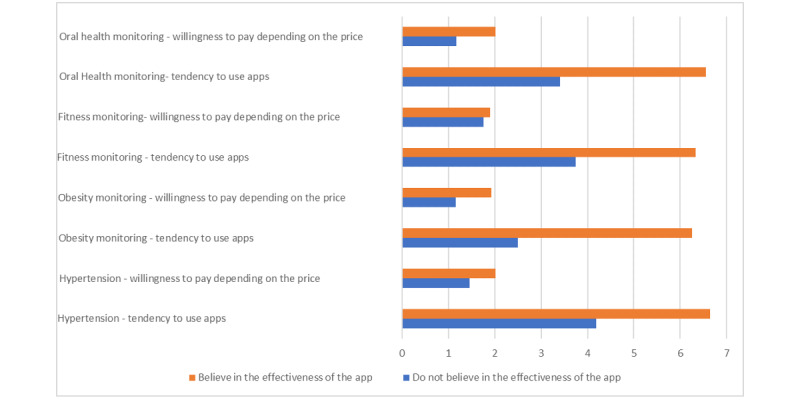
Tendency to use and willingness to pay for medical apps depending on belief in their efficacy.

The final four questions examined which medical field participants would be willing to pay for an app (depending on price) to monitor them:

1. Hypertension: those who believed in the effectiveness of apps to monitor hypertension showed a higher tendency to use an app in this field (mean 6.65, SD 1.44) than those who did not believe in their efficacy (mean 4.19, SD 1.44; *t*_115_=−2.488; *P*=.01; Figure 5). Those who believed (mean 2.01, SD 1.01) in the effectiveness of apps for hypertension showed a higher WTP depending on price than those who did not believe in their efficacy (mean 1.46, SD 0.95; Figure 5; *t*_28.971_=−8.363; *P*=<.001; Figure 5).
2. Obesity: those who believed (mean 6.26, SD 1.13) in the effectiveness of obesity tracking apps showed a higher tendency to use an app in this field than those who did not believe in their efficacy (mean 2.50, SD 1.04; *t*_117_=−1.838; *P*=.04; Figure 5). Those who believed (mean 1.93, SD 0.99) in the effectiveness of obesity tracking apps showed a higher WTP depending on price than those who did not believe in their efficacy (mean 1.16, SD 1.17; *t*_119_=−7.950; *P*<.001; Figure 5).
3. Physical fitness: those who believed (mean 6.33, SD 1.12) in the efficiency of fitness tracking apps showed a higher tendency to use an app in this field than those who did not believe in their efficacy (mean 3.75, SD 1.48; *t*_117_=−7.308; *P*<.001; Figure 5). Interestingly, no statistically significant difference was found between those who believed (mean 1.90, SD 1.02) in the effectiveness of fitness tracking apps and those who did not believe in their efficacy (mean 1.75, SD 0.96) regarding WTP depending on price (*t*_117_=−.505; *P*=.62; Figure 5).
4. Oral health: those who believed (mean 6.56, SD 0.78) in the effectiveness of oral health monitoring apps showed a higher tendency to use an app in this field than those who did not believe in their efficacy (mean 3.41, SD 1.12; *t*_18.739_=−11.125; *P*<.001; Figure 5). Those who believed (mean 2.01, SD 0.97) in the effectiveness of oral health monitoring apps showed a higher WTP depending on price than those who did not believe in their efficacy (mean 1.17, SD 1.01; *t*_115_=−3.257; *P*=.001; Figure 5).

## Discussion

### Principal Findings

The purpose of this mixed methods study was to identify the attitudes, needs, WTP, and perceptions of use in mHealth apps. This study has two sections. The qualitative part included 8 participants of both sexes with different ages and professions. The second, quantitative part, was based on the responses of 121 individuals to a questionnaire sent to them after participation in an 8-week observational study.

The qualitative theme analysis revealed that all the interviewees believed in the ability of a medical app to improve health outcomes in general, and the iGAM app for remote monitoring of gingivitis in particular. The interviewees shared that they do not use medical apps because they believe that they are healthy and have no use for this type of app. Surprisingly, when questioned about their health, most of the interviewees had objective problems, such as hypertension and unbalanced blood glucose. The category analysis showed a contradiction between self-definition and reality.

A systematic review [23] regarding patient perceptions about medical app use found that most participants expressed a positive attitude toward mHealth app use and believed that these apps can promote health. In addition, a survey [24] of 500 physicians and 1000 app users found that 46% of physicians welcomed the medical apps into their lives and believed that they would improve communication between doctors and patients, and 72% of the physicians felt that medical apps would improve patient control over their health. Overall, 96% of the app users believed that medical apps can improve health. Despite the statistically significant belief in the health-promoting abilities of apps, there are barriers preventing app use. Studies [25-29] show that the major issues discouraging medical apps use are related to privacy and security. Other obstacles include app costs and low technological and health literacy [30].

An Australian qualitative study [31] included interviews with 20 general practitioners and 15 older adult patients. They found that among the physicians, the barriers to app use were related to technology, such as fear of prescribing medications through the app, the amount of time required to learn how to use the app, secure storage of private patient information, etc. For patients, the main issues were about ease of use. Interestingly, we discovered a seemingly novel barrier, namely the individual’s definition of themselves as healthy, and therefore not needing a medical app.

The data for the quantitative part of the study came from questionnaires that all participants in our observational study were asked to answer. There were 2 groups in the 8-week observational using the iGAM app. One group took weekly photographs, and the other only took photographs at the beginning and end of the study. The aim of this quantitative study was to understand the perceptions of mHealth app users. 86.8% (105/121) of the survey respondents believed that mobile apps could improve health. Most of the participants were in the third decade of their lives, men, and students. Studies [26,32-34] on the characteristics of medical app users have reported similar findings. The possible selection bias in our study is discussed below (see the *Limitations* section).

A significantly higher tendency to use mHealth apps was found among participants who responded that they first turn to the internet for medical information. These individuals were also willing to pay higher prices for apps than those not using the internet as their first source for clarifying health issues and believed that medical apps could improve health. Consistent with the findings of the qualitative part of the study, most of the respondents noted that they do not currently use mHealth apps but would if they were sick. When asked the general question of whether the participant would be willing to pay for an app related to a disease that they hypothetically have, with no additional information given about the app’s features, a statistically significant majority of both groups, 92.4% (97/105) of the believing group and 75% (12/16) of the nonbelieving group, would use a free app, but as the price rose, the differences between the groups became apparent. The believing group was willing to pay between US $15 (58/105, 55.2%) and US $150% (16/105, 15.2%), whereas in the nonbelieving group, most (9/16, 56%) were not willing to pay US $15, and none agreed to pay US $150.

As we changed the questions slightly, by adding app features making an optimal app, that is, the ability to monitor, offer treatment suggestions, contact the treating physician, and most participants in both groups were willing to pay the maximum amount. This finding contributes to understanding what potential users are looking for in medical apps that they are willing to purchase. WTP increased not only with the tendency to adopt technologies but also with understanding the ability of the app to promote the health of the user. Three components from the HBM [16] can be applied: the perceived benefits, perceived barriers, and cues to action are significant predictors of WTP for mHealth apps.

We found two main reasons for the lack of interest in using medical apps:

- Individuals who prefer an in-person meeting with their physician are less inclined to use applications, and their tendency to pay decreases as the price increases.
- Self-defined as healthy: the participants in this study were relatively young and stated that they were healthy and that this was the reason for the lack of interest in using medical apps. Older populations have more diseases, and younger people tend to be healthier and define themselves as such [35]. Other factors include the findings that different age groups perceive health differently and that older people are more concerned about their health and have more contact with sick friends and family than young people [36]. This may be because younger people are preoccupied with matters, such as starting a family, establishing a career, etc.

When we examined the willingness to use and pay for apps that monitor hypertension, obesity, and physical fitness, those who believe that apps can improve health had a significantly higher propensity to use an app for all these issues. However, when examining WTP, the results were different. A statistically significant difference in WTP was only found for monitoring hypertension, obesity, and oral health monitoring, and no significant difference was found for fitness tracking. This may be because of the fact that most smartphones come with free fitness–related apps [37,38]; therefore, people may be reluctant to spend money on another similar app.

### Limitations

The main limitation of this study is the difficulty of generalizing the results because of a possible selection bias among the volunteer participants. First, the participants were young, and perceptions of wellness and health may be different in older age groups. Second, most of the participants were students who may be more comfortable with technology and have a certain level of health and technological literacy. These issues can be resolved by including individuals of different ages in the study groups.

It is possible that there was a selection bias regarding the characteristics of the interviewees (4 out of 8 were involved in the health sector: 1 physician, 2 nurses, and 1 dental student). Therefore, we decided that all study participants should complete the quantitative questionnaire. Nevertheless, there is a need for further research to examine the acceptance of iGAM mHealth apps in groups with different levels of literacy. Another potential limitation is that the qualitative data were coded by one person; therefore, to increase credibility, an independent expert examined the process of the entire data analysis and themes.

The fact that all participants defined themselves as healthy is a substantial limitation of this study. The questionnaire may not have been sensitive enough, and we may need to include individuals that classify themselves as *sick* or *unwell* or those taking medication regularly, for example, those treated for high blood pressure or diabetes. However, we follow the broad definition of health of the World Health Organization, namely “Health is a state of complete physical, mental and social well-being and not merely the absence of disease or infirmity,” and assumed that our qualitative and quantitative methods gave the participants the leeway needed to define their own health status and yield results that accurately represent their attitudes toward the use of medical apps.

### Conclusions

This study found that people believe an mHealth app can be used to monitor gingivitis to improve gum health. Furthermore, people are willing to use and pay for an mHealth app depending on their perceptions of their health requirements, state of health, and the level of active involvement of the app promoting health for the particular disease they have.

## Appendix

Multimedia Appendix 1 iGAM interview plan.

Multimedia Appendix 2 Characteristics of the study population.

## Abbreviations

HBM: Health Belief Model
NIS: New Israeli Shekel
RPM: remote patient monitoring
WTP: willingness to pay
