# Supplementary material for: Perceptions and Attitudes Toward the Use of a Mobile Health App for Remote Monitoring of Gingivitis and Willingness to Pay for Mobile Health Apps (Part 3): Mixed Methods Study
Source: JMIR Form Res. 2021 Oct 5;5(10):e26125. doi: 10.2196/26125 (PMC8527382; doi:10.2196/26125)
Supplement: Multimedia Appendix 1 [file formative_v5i10e26125_app1.docx]

Appendix 1: iGAM Interview plan (Mixed Methods Study; Qualitative arm)

IGAM Interview Program

Opening questions

1. How would you describe your needs from a mobile phone?
2. Where does the cell phone fit into your daily life?
3. How would you describe the uses you make of the cell phone?
4. Do these uses differ as much from the first phone to the one you use today?

Questions about mobile applications –

1. Describe your interest in mobile applications
2. Describe the applications currently installed on your phone?
3. How often do you use these applications?
4. What is your perception ofthe use of medical applications?
5. What medical applications do you know?
6. What is the purpose of using a medical application?
7. Can you imagine using a medical application to enhance health?

Questions regarding the iGAM study

1. Please describe your expectations of the app before using it?
2. Describe to me your impression of the study?
3. Describe your experience using the app?
4. How did you perceive the application to affect you, and in what way?
5. Who do you think would be interested in such an application?
6. Is there a change in the state of your mouth as a result of using the app?
7. Do you perceive the feedback you received as credible?
8. Has the feedback led you to change your behavior?
9. Would you change (add or subtract) features in the app

Closed questions
- Age_____
-Residence_____

-Personal and professional status______

Do you have anything to add?
