# Supplementary material for: Perceptions and Attitudes Toward the Use of a Mobile Health App for Remote Monitoring of Gingivitis and Willingness to Pay for Mobile Health Apps (Part 3): Mixed Methods Study
Source: JMIR Form Res. 2021 Oct 5;5(10):e26125. doi: 10.2196/26125 (PMC8527382; doi:10.2196/26125)
Supplement: Multimedia Appendix 2 [file formative_v5i10e26125_app2.docx]

**Multimedia Appendix.** Characteristics of the study population.

| Group 2^b^: apps can improve health, n (%) | Group 1^a^: apps cannot improve health, n (%) | Research questions | | | | |
| --- | --- | --- | --- | --- | --- | --- |
| **When I need to find medical information, the sources I use are** | | | | | | |
| **Written press or medical** | | | | |  |  |
| 22 (21) | 5 (31.3) | Agree |  | | |  |
| 83 (79) | 11 (68.8) | Disagree |  | | |  |
| **Internet sites** | | | | |  |  |
| 93 (88.6) | 13 (81.3) | Agree |  | | |  |
| 12 (11.4) | 3 (18.8) | Disagree |  | | |  |
| **Friends or family** | | | | |  |  |
| 50 (47.6) | 5 (31.3) | Agree |  | | |  |
| 55 (52.4) | 11 (68.8) | Disagree |  | | |  |
| **Contacting a medical professional** | | | | |  |  |
| 63 (60) | 11 (68.8) | Agree |  | | |  |
| 42 (40) | 5(31.3) | Disagree |  | | |  |
| **When I need medical information about myself, I search the internet first** | | | | | | |
| 90 (85.7) | 12 (75) | Agree | | | |  |
| 15 (14.3) | 4 (25) | Disagree | | | |  |
| **I use mobile applications to improve my health** | | | | | | |
| 35 (33.3) | 2 (12.5) | Use | | | |  |
| 70 (66.7) | 14 (87.5) | Don’t use | | | |  |
| **If so, what type of app are you using?** | | | | | | |
| 70 (66.  7) | 14 (87.5) | Does not use mobile applications | | | |  |
| Missing data | Missing data | Exercise apps | | | |  |
| 13 (12.4) | Missing data | App with tools for my specific medical problem | | | |  |
| **If you answered yes to the question above, why do you use it?** | | | | | | |
| 4 (3.8) | 0 | I have a specific medical problem and the app is free | | | |  |
| 18 (17.1) | 2 (12.5) | Other | | | |  |
| 68 (64.8) | 14 (87.5) | Does not use | | | |  |
| **If I were sick with a chronic illness, I would download a free medical app related to the disease** | | | | | | |
| 97 (92.4) | 12 (75) | Agree | | | |  |
| 8 (7.6) | 4 (25) | Disagree | | | |  |
| **If I were sick with a chronic illness, I would download a 50 NIS (one-time fee) medical app related to the disease** | | | | | | |
| 58 (55.2) | 7 (43.8) | Agree | | | |  |
| (44.8) 47 | (56.3) 9 | Disagree | | | |  |
| If I were sick with a chronic illness, I would download a 500 NIS (one-time fee) medical app related to the disease | | | | | | |
| 16 (15.2) | 0 | Agree | | | |  |
| 89 (84.8) | 16 (100) | Disagree | | | |  |
| **If I were sick with a chronic illness, I would pay a maximum amount of? NIS (one-time fee) for an optimal medical app (see below for details)** | | | | | | |
| 4 (3.8) | 1 (6.3) | $3 | | | |  |
| 22 (21) | 2 (12.5) | $15 | | | |  |
| 48 (45.7) | 7 (43.8) | 0$3 | | | |  |
| 30 (28.6) | 6 (37.5) | I have no interest in such apps | | | |  |
| **If you answered that you have no interest in medical applications, what is the reason for this?** | | | | | | |
| 3 (2.9) | 1 (6.3) | I’m afraid my personal information will be collected | | | |  |
| 0 | 0 | Do not believe these apps are reliable | | | |  |
| 3 (2.9) | 1 (6.3) | Prefers the existing situation | | | |  |
| 26 (24.8) | 4 (25) | I’m healthy so I do not need medical apps | | | |  |
| 0 | 0 | Not interested in answering | | | |  |
| 0 | 0 | Other | | | |  |
| 73 (69.5) | 10 (62.5) | I have an interest in medical apps | | | |  |
| **I tend to believe apps can effectively monitor:** | | | | | | |
| **Hypertension** | | | |  |  |  |
| 16 (15.2) | 6 (37.5) | Agree |  | | |  |
| 85 (81) | 10 (62.5) | Disagree |  | | |  |
| **Obesity** | | | |  |  |  |
| 100 (95.2) | 13 (81.3) | Agree |  | | |  |
| 3 (2.9) | 3 (18.8) | Disagree |  | | |  |
| **Fitness** | | | |  |  |  |
| 96 (91.4) | 11 (68.8) | Agree |  | | |  |
| 7 (6.7) | 5 (31.3) | Disagree |  | | |  |
| **Oral health** | | | |  |  |  |
| 93 (88.6) | 7 (43.8) | Agree |  | | |  |
| 8 (7.6) | 9 (56.3) | Disagree |  | | |  |
| **Regarding my general state of health, I see myself as:** | | | | | | |
| 105 (100) | 16 (100) | Healthy | | | |  |
| — | — | Not healthy | | | |  |
| **Regarding my oral health status, I see myself as:** | | | | | | |
| 105 (100) | 16 (100) | Healthy | | | |  |
| — | — | Not healthy | | | |  |

^a^Group 1: lack of faith that mobile apps can improve health.

^b^Group 2: belief that mobile apps can improve health.
